# Why people listen: Motivations and outcomes of podcast listening

**DOI:** 10.1371/journal.pone.0265806

**Published:** 2022-04-06

**Authors:** Stephanie J. Tobin, Rosanna E. Guadagno

**Affiliations:** 1 School of Psychology and Counselling, Queensland University of Technology, Brisbane, Queensland, Australia; 2 Center for International Security and Cooperation, Stanford University, Stanford, California, United States of America; Universitat Luzern, SWITZERLAND

## Abstract

The aim of this preregistered study was to identify dispositional predictors of podcast listening and examine the associations between aspects of podcast listening, dispositional predictors, and psychological outcomes. Three hundred and six adults from a range of countries completed an online questionnaire that assessed individual difference predictors (the Big Five personality factors, curiosity, need for cognition, need to belong, age, and gender), aspects of podcast listening (amount, format, setting, device, and social aspects), and potential outcomes (autonomy, competence, relatedness, meaning, mindfulness, and smartphone addiction). As predicted, openness to experience, interest-based curiosity, and need for cognition positively predicted podcast listening. Contrary to predictions, need to belong negatively predicted podcast listening, and time spent listening to podcasts was not associated with autonomy, competence, relatedness, meaning, mindfulness, or smartphone addiction. However, certain aspects of podcast listening (e.g., parasocial relationships and social engagement) were related to positive outcomes and to our predictor variables. Furthermore, neuroticism negatively predicted podcast listening. Overall, the findings support the idea that informational motives can play a role in podcast listening, and that some aspects of listening are associated with positive outcomes.

## Introduction

Podcasts, audio recordings that are played on demand usually from people’s smart phones, have risen in popularity in recent years, with the estimated number of podcast listeners rising from 46.1 to 75.9 million in the US and from 8.99 to 15.61 million in the UK from 2017–2020 [1,2]. Across 20 countries, 31% of survey respondents reported having listened to a podcast in the last month [3]. Scholars have analyzed the popularity of podcasts, noting the similarity to radio in terms of being an audio medium and highlighting the high levels of intimacy, sociality, and flexibility that podcast listening affords [4,5]. Thus, podcasting has become a modern-day radio with a wide variety of news, information, interviews, and stories, both fact and fiction, to select and listen to on demand.

While a number of studies have examined podcast listening in an educational context [6,7], research on more mainstream podcast listening is in its infancy [5]. Several of these early studies have adopted a uses and gratifications perspective to identify reasons for listening to podcasts [8–11]. For instance, a survey of regular podcast listeners in the US revealed that the strongest motives for listening were entertainment, information, and audio platform superiority [11]. Other research revealed that social engagement, edutainment, and storytelling gratifications were significant predictors of podcast use [8,10]. Furthermore, a qualitative study of podcast listeners found that people often listen to podcasts while completing mundane tasks such as commuting or household chores, which enriched their experience and left participants feeling productive and that they learned something new [9]. These podcast listeners also reported feeling connected with podcast hosts and fellow listeners [9].

Consistent with the uses and gratifications paradigm [12], past research on podcast listening has focused on people who listen to podcasts and asked them to discuss or rate their motivations for listening [8–11]. However, there are some limitations to this approach. First, this approach assumes that people are aware of their motives [12]. Second, it tends to generate a large set of media-specific motives without necessarily tapping into deeper psychological needs [12]. An alternative approach that addresses these limitations is to assess broader dispositional factors and examine whether they predict media use [13–16].

### Dispositional predictors of podcast use

The differential susceptibility to media effects model is a broad, integrative model that specifies various pathways by which susceptibility factors and media use can impact people’s thoughts, feelings, and behaviors [13]. According to this model, media effects depend on dispositional, developmental, and social factors, which influence the selection of and reactions to media. The lasting effects of media on thoughts, feelings, and behaviors depend on response states that occur during media use, and media effects can affect subsequent susceptibility, media use, and response states. Media properties (e.g., modality, content, structure) can also play a role in media effects [17]. In applying this model to podcast listening, we draw upon early research on podcast listening and related work on the adoption of new technologies and use of entertainment media to identify relevant dispositional factors that may increase attraction to podcasts.

Past research on motives for listening to podcasts has revealed that podcasts provide listeners with both informational and social gratifications [8–11]. Accordingly, we identified individual differences relevant to informational (i.e., openness to experience, curiosity, and need for cognition) and social (i.e., need to belong) needs. Assessing dispositional factors outside the context of podcast listening allowed us to compare the dispositions of people who listened to podcasts to those who did not [for a similar approach, see 14,15].

#### Informational needs

The Big Five approach to personality focuses on individual differences in extraversion, neuroticism, openness to experience, agreeableness, and conscientiousness [18]. Openness to experience is the dimension most closely aligned with informational needs. It describes the extent to which individuals have a broad, deep, original, and complex mental and experiential life [19]. Podcasts should appeal to those high in openness to experience because they allow people to explore and pursue a range of topics. Past research has found that openness to experience is associated with use of Twitter, an informationally oriented social media platform [14], and early adoption of blogging [20]. With podcasting still in its nascence and linked to informational motives, we expected people high in openness to experience to once again be early adopters of this new technology.

We did not have directional predictions for the other Big Five personality traits, but we examined them to build a more complete profile of podcast listeners. Extraversion includes sociability and activity, neuroticism involves negative emotionality, agreeableness involves prosocial and communal views of others, and conscientiousness involves impulse control and goal-directed behavior [19]. The Big Five personality traits have been studied extensively in relation to social media use [for reviews see 21,22]. Facebook users have been found to be more extraverted and less conscientious than nonusers [15]. Furthermore, a meta-analysis revealed that amount of social media use was positively associated with extraversion and neuroticism, negatively associated with conscientiousness, and not significantly associated with openness or agreeableness [21]. However, podcasts differ from social media in many ways (e.g., modality, content, affordances), so the associations might differ.

Curiosity is a component of openness to experience but also a complex construct of its own. We focused on two types of epistemic curiosity: interest and deprivation [23]. Epistemic curiosity refers to the desire for knowledge, with interest reflecting an enjoyment of new discoveries and deprivation reflecting a discomfort with not knowing [23]. Researchers have argued for the relevance of epistemic curiosity for understanding different types of engagement in online communities, positing that interest-based curiosity should predict more passive engagement, or lurking, due to greater enjoyment of new information [24]. Similarly, curiosity about people was found to be positively associated with use of social networking sites [25]. We expected interest-based curiosity to positively predict podcast listening, as podcasts offer many new discoveries.

Need for cognition reflects enjoyment of and engagement in effortful cognitive endeavors [26]. Need for cognition has been found to positively predict using the internet for product information, news, and learning [27] and using Twitter for informational purposes [28]. In terms of other types of media use, college students’ need for cognition levels were positively associated with reading non-fiction books and novels, negatively associated with watching television, and not significantly associated with reading or writing blogs, using social media, or messaging [29]. Podcasts should appeal to those high in need for cognition, as they would allow them to “feed the brain” while engaging in mundane tasks [9].

#### Social needs

Prior research indicates that podcasts can serve a social function [8–11], so they might appeal to people with a high need to belong [30]. Although all people are thought to have a basic need to belong which is best met through regular positive interactions in the context of ongoing relationships [31], some people have stronger desires for acceptance and belonging than others [30]. People with a higher need to belong report more addictive social networking site tendencies [32]. They also form stronger parasocial relationships with their favorite TV personalities [33]. Podcasts offer informal and intimate conversation [5], which could be attractive to those with a high need to belong.

### Aspects of podcast use

Past research on podcast listening has examined characteristics of podcast use such as amount and setting and found that they relate to age and gender. Chan-Olmsted and Wang (11) found that men listen more widely and deeply than women, and younger people listen more widely, deeply, and routinely. They also found that women relative to men were more likely to listen to podcasts at home and less likely to listen at work, while younger people were less likely to listen at home and more likely to listen at work or on the go [11]. This approach provided a more detailed understanding of how different types of users engage with podcasts and went beyond earlier research which had found that men were more likely than women to listen to podcasts and age did not predict podcast listening [34]. Accordingly, we examined age and gender as predictors of podcast listening and various aspects of podcast use.

In addition to the amount and setting of podcast listening, we examined device used to listen, podcast format, and social aspects of podcast listening. Similar to setting, device could relate to whether people are listening to podcasts while engaged in other activities. With format, we were interested in the potential for conversation between multiple hosts or between host(s) and guest(s), as well as the potential for pre-existing attachments to hosts. These features could help listeners feel like they are part of the conversation and connect with the host(s) [9,35].

Previous research has found that social aspects of podcast listening include feeling connected to hosts and fellow listeners [8–11,35]. Feeling connected to a podcast host can lead to a parasocial relationship: a one-sided relationship people form with a media figure or celebrity [36,37]. Research has found that podcast listeners form stronger parasocial relationships with their favorite podcast host when the host shows an interest in listeners, shares personal information, and is seen as more competent, authentic, and unpredictable [36]. A recent experiment further revealed that greater self-disclosure by a podcast guest increased perceived levels of parasocial interaction among listeners [38]. Lastly, a qualitative study of successful journalism podcasts revealed they used a personal and subjective narrative style to foster a sense of intimacy and emotional engagement among listeners [39]. Overall, the emerging literature on parasocial relationships between podcasters and their listeners suggests that people can form such attachments and that a podcaster’s communication style can affect the strength of the parasocial relationship.

For all aspects of podcast listening (i.e., amount, setting, format, device, social), we examined associations with our individual differences related to informational and social needs. This would allow us to understand whether different motives for listening were associated with different ways of engaging with podcasts.

Social engagement and parasocial relationships may be more likely to develop with extended listening, so we also examined associations between these variables and other aspects of podcast listening. Past studies have found that greater media consumption was associated with stronger parasocial relationships [36,40], stronger parasocial interaction perceptions during a podcast were associated with stronger intentions to subscribe and continue engaging with a podcast [38], and higher social engagement with podcasts was associated with downloading more podcasts per week [8].

### Potential outcomes of podcast listening

Research has revealed that entertainment media can directly or vicariously satisfy basic psychological needs for autonomy, competence, and relatedness [41]. Autonomy refers to experiences of agency and volition and can be satisfied when people endorse and value their actions. Competence involves feeling a sense of efficacy and personal growth and can be satisfied by learning new things, feeling mastery, and succeeding in challenges. Relatedness involves feeling socially connected and can be satisfied when people feel cared for by others and significant to others [42]. According to Self Determination Theory, satisfying autonomy, competence, and relatedness needs is essential for psychological health, well-being, and a sense of meaning in life [42].

Podcast listening could help people satisfy their basic psychological needs. Podcast listeners are free to choose from over 2 million different podcasts [43] and can decide when and where they listen to them [44]. As such, podcast listening might enhance one’s sense of autonomy. With low barriers to production and distribution, and little regulation or constraint [4,5,44], many different podcasts are freely available to listeners. Podcasts provide a relatively easy way to increase one’s knowledge about a variety of topics, so podcast listening could potentially enhance one’s sense of competence. The informal and intimate style of some podcasts and of the listening experience [4,5,44,45] may lead to the formation of parasocial relationships with podcast hosts [9,36,38,46]. Further, listeners can directly interact with podcast hosts and fellow listeners through social media platforms and form richer, more genuine relationships than the parasocial relationships afforded by earlier forms of entertainment media [5,8,9]. These social aspects of podcast listening might enhance listeners’ sense of relatedness.

Could podcast listening also contribute to a sense of meaning in life? Generally, meaning in life includes a sense of purpose, significance, and coherence [47]. Meaning in life is associated with the satisfaction of basic psychological needs, positive affect, environmental coherence, and curiosity [47–49]. Research has revealed that videogames viewed as meaningful have stronger narrative elements, which in turn predict a greater sense of relatedness, insight, and autonomy [50]. To the extent that podcasts contain narrative elements, satisfy needs, stimulate curiosity and positive affect, and/or provide a sense of coherence, we would expect listening to be associated with a greater sense of meaning in life.

As with any new technology, it is important to consider the potential negative outcomes of podcast listening alongside the positive outcomes. Podcast listening often occurs via smartphones and while people are engaged in other activities [9,51]. Research has revealed that more frequent use of smartphone features including music/podcasts/radio was associated with higher levels of smartphone addiction [52]. Smartphone addiction involves phone-related disturbances to daily life, withdrawal, overuse, tolerance, online relationships, and positive anticipation [53]. Because podcast listening often occurs on smartphones, it could contribute to smartphone addiction.

Furthermore, research has found that higher levels of media multitasking were associated with lower levels of mindful attention awareness [54]. This form of mindfulness reflects a present-centered awareness in daily life [55]. Listening to podcasts while engaging in other activities would likely divert attention away from the other activities, reducing one’s present-centered awareness.

### The current research

The current study aimed to identify dispositional predictors of podcast listening and examine the associations between aspects of podcast listening, dispositional predictors, and psychological outcomes. We recruited a broad sample that included both those who had and had not listened podcasts. All participants completed measures of our dispositional and outcome variables of interest, and those who indicated that they had ever listened to a podcast completed additional questions about their podcast use. All hypotheses and research questions except for those involving age and gender, and the associations between social and other aspects of podcast use were preregistered prior to conducting the research at https://osf.io/kp4dw. Our materials and data are available online at https://osf.io/dwcaz/.

## Method

### Participants

At the time we designed our study, we used the 2018 estimate that 44% of Americans aged 12 and older have ever listened to a podcast [51]. We aimed to recruit a general sample of 300 participants in order to obtain approximately 132 podcast listeners (44% of 300). A sensitivity analysis in G*Power revealed this would give us .80 power to detect correlations as small as .24 in our sample of podcast listeners [56]. We advertised our study on Prolific (https://www.prolific.co/), a crowdsourcing platform for academic research that has been shown to provide a more honest and naïve participant pool than other options such as Amazon’s Mechanical Turk [57]. Although we set the maximum number of submissions to 300, we had 308 participants complete study due to some participants timing out in the Prolific system and not counting as a submission. Two participants opted to withdraw their data and were not included in any analyses. Each participant was paid £2.50 ($3.25 USD) for the 30-minute study.

### Measures

#### Demographic and predictor variables

Participants were asked to indicate their age, gender, and country of residence.

The Big Five Inventory [18] contains 44 items. Participants indicated the extent to which they saw themselves as someone who possesses a variety of characteristics on a 5-point scale (1 = *disagree strongly*, 5 = *agree strongly*). The items assess their levels of extraversion (e.g., “is talkative,” α = .85), agreeableness (e.g., “is helpful and unselfish with others,” α = .74), conscientiousness (e.g., “does a thorough job,” α = .79), neuroticism (e.g., “is depressed, blue,” α = .84), and openness to experience (e.g., “is original, comes up with new ideas,” α = .76).

The interest/deprivation scale [23] contains 10 items rated on a 4-point scale (1 = *almost never*, 4 = *almost always*). It assesses participants’ levels of interest (e.g., “I enjoy exploring new ideas,” α = .79) and deprivation (e.g., “difficult conceptual problems can keep me awake all night thinking about solutions,” α = .83), two types of epistemic curiosity.

The short version of the need for cognition scale [58] contains 18 items rated on a 5-point scale (1 = *extremely uncharacteristic*, 5 = *extremely characteristic*). It assesses the extent to which individuals engage in and enjoy thinking (e.g., “I prefer complex to simple problems,” α = .89).

The need to belong scale [30] contains 10 items rated on a 5-point scale (1 = *not at all*, 5 = *extremely*). It assesses the strength of individuals’ belonging needs (e.g., “I try hard not do things that will make other people avoid or reject me,” α = .83).

#### Podcast listening variables

Participants were first asked if they had ever listened to a podcast (yes or no). Those who said yes were then asked a series of follow up questions regarding their podcast listening habits.

Participants indicated how often they listen to podcasts (daily, weekly, monthly, less than monthly), how many years they have been listening to podcasts, how many hours per week they spend listening to podcasts, and how many different podcasts they listen to in a typical month.

Participants selected the type of podcasts they typically listen to from a list of iTunes podcast categories (arts, business, comedy, education, games & hobbies, government & organizations, health, kids & family, music, news & politics, religion & spirituality, science & medicine, society & culture, sports & recreation, technology, TV & film, other).

Participants indicated the percentage of podcasts (of all the ones they listen to) that have a single host, have more than one host, are hosted by people they knew from somewhere else (e.g., actors or comedians), and interview guests.

Participants indicated the percentage of time (of all time spent listening to podcasts) they listen to podcasts while engaged in another activity at home, engaged in another activity away from home, and not engaged in any other activity.

Participants indicated the percentage of time (of all time spent listening to podcasts) they listen to podcasts on a smartphone, on a mobile device other than a smartphone, and on a computer.

We examined general podcast-related social engagement and specific parasocial relationships with one’s favorite podcast host. For general social engagement, participants rated on a 7-point scale (1 = *not at all*, 7 = *to a great extent*) the extent to which they followed podcasts on social media, discussed podcasts with other people, and felt connected to other people who listen to the same podcasts. We averaged these three items to create an index of social engagement with podcasts (α = .71).

We adapted the parasocial interaction scale [59] so that it referred to a favorite podcast host rather than favorite TV personality. Participants were first asked to identify their favorite podcast host and then rate the extent to which they agree with 15 items (e.g., “I think my favorite podcast host is like an old friend,” α = .93) on a 5-point scale (1 = *strongly disagree*, 5 = *strongly agree*).

#### Outcome variables

The basic psychological need satisfaction scale [60,61] contains 21 items rated on a 7-point scale (1 = *not at all true*, 7 = *very true*). It assesses the extent to which participants are meeting their needs for autonomy (e.g., “I feel like I am free to decide for myself how to live my life,” α = .72), competence (e.g., “People I know tell me I am good at what I do,” α = .76), and relatedness (e.g., “I really like the people I interact with,” α = .83).

The meaning in life questionnaire [62] contains 10 items that are rated on a 7-point scale (1 = *absolutely untrue*, 7 = *absolutely true*). It assesses the presence of (e.g., “I understand my life’s meaning,” α = .90) and search for (e.g., “I am looking for something that makes my life feel meaningful,” α = .88) meaning in life.

The mindful attention awareness scale [55] contains 15 items that are rated on a 6-point scale (1 = *almost always*, 6 = *almost never*). It assesses the extent to which participants pay attention to and are aware of current internal and external events (e.g., “I could be experiencing some emotion and not be conscious of it until some time later,” α = .88).

The short version of the smartphone addiction scale [63] contains 10 items that are rated on a 6-point scale (1 = *strongly disagree*, 6 = *strongly agree*). It assesses the extent to which participants endorse signs of smartphone addiction such as disturbance of daily life, overuse, tolerance, cyberspace-oriented relationships, and withdrawal (e.g., “Feeling impatient and fretful when I am not holding my smartphone,” α = .86).

### Procedure

This research was approved by the Human Research Ethics Committee at Queensland University of Technology. The data were collected between July 31 and August 3 in 2019. After reading the information sheet, participants were asked to indicate their consent by clicking "I consent, begin the study" and those who consented completed the online study in Qualtrics. The order of questionnaire was randomized. Only participants who indicated they had ever listened to a podcast (*n* = 240) received the podcast-related questions. At the end of the study, all participants received an online debriefing and were given a chance to enter comments and withdraw their data.

### Data analysis

In the full sample, we ran logistic regressions to examine continuous predictors of podcast listening and a chi-squared test to examine the association between gender and podcast listening. In the sample of podcast listeners, we examined correlations between aspects of podcast listening, dispositional factors, and psychological outcomes. Because of the large number of correlations, we set the false discovery rate to .05 to control type I error [64].

## Results

After reverse scoring the appropriate items, scale scores were calculated by averaging the relevant items for each scale, provided that participants had answered at least half of the items. Outlying values more than 3 SD from the mean were recoded to 3 SD from the mean to retain the data but reduce its influence [65].

### Descriptive statistics

The final sample consisted of 157 male, 146 female, and 2 other gender participants, with missing gender data for 1 participant. Ages ranged from 18–64 (*M* = 27.87, *SD* = 9.16). Participants were from a variety of countries including the UK (22%), USA (14%), Portugal (14%), Poland (10%), Canada (8%), Mexico (6%), Greece (6%), Spain (4%), Italy (3%), Australia (2%), and others (< 2% each).

Among our 306 participants, 240 (78.43% of our sample) reported that they had listened to a podcast, while 66 had not. Of those who had listened to a podcast, 106 listened less than monthly, 32 listened monthly, 72 listened weekly, and 30 listened daily. The most frequently selected categories of podcasts were comedy (48%), games and hobbies (34%), society and culture (23%), music (23%), news and politics (23%), and education (21%). Descriptive statistics for the other variables are presented in Table 1. On average, our podcast listeners had been listening for 3 years, for 3.5 hours per week, and to 3 different podcasts per month. All formats (i.e., single/multiple hosts, known hosts, interview guests) were popular. Most participants listened to podcasts while engaging in other activities at home and most listened on a smartphone. Social engagement with podcasts was relatively low overall, although parasocial relationships were moderate.

**Table 1:** Descriptive statistics.

	Mean	St Dev
**Predictor Variables**		
Extraversion	2.79	0.82
Agreeableness	3.54	0.63
Conscientiousness	3.34	0.65
Neuroticism	3.28	0.83
Openness	3.58	0.63
Curiosity-interest	3.04	0.63
Curiosity-deprivation	2.58	0.71
Need for Cognition	3.31	0.67
Need to Belong	3.06	0.76
**Podcast Listening Variables**		
Amount		
Hours/week	3.55	5.13
Years	3.17	2.68
Podcasts/month	3.15	3.11
Format		
% Single host	47.99	36.77
% Multiple hosts	51.94	36.82
% Known host	47.85	39.31
% Interview guests	49.69	34.11
Setting		
% Activity-home	58.10	32.60
% Activity-away	36.76	35.03
% Activity-none	29.42	30.36
Device		
% Device-smartphone	62.25	35.96
% Device-other	13.82	24.86
% Device-computer	45.48	36.98
Social Aspects		
Parasocial relationship	3.32	0.86
Social engagement	3.02	1.54
**Outcome Variables**		
Autonomy	4.56	0.95
Competence	4.44	1.12
Relatedness	4.83	1.06
Meaning-Presence	3.97	1.44
Meaning-Search	4.99	1.22
Mindfulness	3.66	0.87
Smartphone Addiction	2.76	1.04

### Predicting podcast listening

Because of the conceptual overlap between openness, curiosity, and need for cognition (*r*s ranged from .35 to .65, *p*s, < .001), we examined each predictor in a separate logistic regression (see Table 2). As predicted, openness, interest-based curiosity, and need for cognition were significant positive predictors of podcast listening. Deprivation curiosity was not a significant predictor. Contrary to predictions, people with a higher need to belong were significantly less likely to have listened to a podcast.

**Table 2:** Logistic regressions predicting podcast listening.

Omnibus model							Predictors				
Model #	χ^2^	df	N	p	Cox and Snell *R*^2^	Nagelkerke *R*^2^	Variable	B	Exp (B)	p	95% CI
1	4.22	1	306	.040	.01	.02	Openness	.45	1.57	.040	1.02, 2.42
2	6.39	2	306	.041	.02	.03	Curiosity-interest	.65	1.91	.013	1.15, 3.17
							Curiosity-deprivation	-.35	0.71	.126	0.45,1.10
3	5.85	1	306	.016	.02	.03	Need for Cognition	.51	1.66	.016	1.10, 2.50
4	4.06	1	306	.044	.01	.02	Need to Belong	-.38	0.69	.046	0.48, 0.99
5	13.51	5	306	.019	.04	.07	Openness	.50	1.65	.043	1.02, 2.69
							Neuroticism	-.52	0.59	.008	0.40, 0.87
							Extraversion	-.17	0.85	.396	0.58, 1.24
							Agreeableness	.08	1.08	.739	0.68, 1.73
							Conscientiousness	-.44	0.64	.074	0.39, 1.04
6	1.12	1	304	.291	< .01	.01	Age	-.02	0.98	.284	0.96, 1.01

An additional logistic regression model with all Big Five factors as predictors of having listened to a podcast was significant (see Table 2). Openness remained a significant positive predictor, neuroticism was a significant negative predictor, and extraversion, agreeableness, and conscientiousness were not significant predictors.

A chi-square test indicated that male participants (84%) were significantly more likely than female participants (73%) to have listened to a podcast, χ^2^(1, *N* = 303) = 5.29, *p* = .022. However, a logistic regression revealed that age did not predict podcast listening (see Table 2).

### Associations among podcast listeners

An examination of the correlations between the podcast variables and individual difference predictor variables (see Table 3) revealed that older participants had been listening for more years. Furthermore, extraversion was positively associated with social engagement and agreeableness was positively associated with parasocial relationships.

**Table 3:** Correlations between the podcast variables and individual difference predictor variables.

	Extraversion	Agreeableness	Conscientiousness	Neuroticism	Openness	Curiosity-interest	Curiosity-deprivation	Need for Cognition	Need to Belong	Age	Gender
Hours/week	-.05	-.06	.05	.02	.01	.06	.00	.05	-.02	-.02	.03
Years	.04	.01	.08	-.09	.10	.07	-.06	.19	-.18	.22*	-.11
Podcasts/month	.05	.06	.15	.01	.11	.13	.02	.09	-.07	.07	-.01
Single host	.00	-.01	.03	.01	.01	.00	.00	-.04	.03	.11	.06
Multiple hosts	-.02	-.01	-.12	.01	-.03	.02	-.07	.03	-.03	-.16	-.06
Known host	.00	.09	-.10	.06	-.07	-.04	-.07	-.10	.03	-.20	.01
Interview guests	.11	.02	.07	.00	.00	.04	-.11	.16	.03	.05	.04
Activity-home	.04	-.03	-.06	.06	.04	.07	.00	-.01	.04	-.18	.11
Activity-away	-.01	-.08	-.12	.01	.06	.02	.05	.07	-.02	-.13	-.02
Activity-none	.07	.04	-.03	-.11	.03	-.01	.06	-.04	.07	.16	-.17
Device-smartphone	-.04	-.03	-.04	.09	.04	.09	.01	-.03	.10	-.13	.15
Device-other	.19	.08	.02	-.06	.01	-.07	.07	.09	-.13	.06	-.01
Device-computer	.03	-.08	-.04	-.12	-.04	-.07	-.06	-.03	-.13	.01	-.15
Parasocial relationship	.10	.22*	.15	.00	.09	.15	.12	-.05	.06	.01	-.04
Social engagement	.28*	.12	.14	.01	.07	.10	.09	.01	.06	.02	.00

*Note*. * indicates significance with the false discovery rate set at .05. Gender is coded 1 = female, 0 = male.

An examination of the correlations between the social and other aspects of podcast listening (see Table 4) revealed that parasocial relationships and social engagement were positively associated with each other. Social engagement was associated with listening for longer and to a greater number of different podcasts. Parasocial relationships were associated with listening for longer and listening to podcasts by known hosts.

**Table 4:** Correlations between the podcast variables and potential outcome variables.

	Autonomy	Competence	Relatedness	Meaning-Presence	Meaning-Search	Mindfulness	Smartphone Addiction	Parasocial relationship	Social engagement
Hours/week	.03	.06	-.04	.07	.03	.05	-.04	.20*	.24*
Years	.15	.15	.03	.14	-.13	.10	-.21*	.05	.12
Podcasts/month	.12	.11	.05	.21*	.13	.00	-.06	.17	.27*
Single host	-.01	-.02	-.04	-.02	-.07	-.07	.08	-.10	.02
Multiple hosts	-.01	-.08	.02	-.06	.11	.04	-.03	.13	.01
Known host	.01	-.10	.15	-.14	.03	.00	.13	.23*	.12
Interview guests	.05	-.05	.03	-.03	-.03	.03	.11	.13	.13
Activity-home	-.10	-.04	-.05	-.11	.09	-.03	.05	-.08	-.02
Activity-away	-.06	-.01	-.04	-.05	-.04	-.18	.14	-.01	-.05
Activity-none	.18	.13	.13	.16	-.13	.15	-.10	.07	.08
Device-smartphone	-.06	-.01	.04	-.07	.07	-.15	.19	.01	-.03
Device-other	.01	.05	.05	-.02	-.01	.08	.11	.11	.17
Device-computer	.00	-.01	-.08	-.01	.02	.12	-.16	-.07	-.06
Parasocial relationship	.08	.08	.24*	.16	.19	.00	.08		
Social engagement	.10	.15	.19	.20*	.09	.08	.13	.46*	

Note. * indicates significance with the false discovery rate set at .05.

The correlations between the podcast listening and outcome variables (see Table 4) indicated that contrary to predictions, hours per week spent listening to podcasts was not significantly correlated with autonomy, competence, relatedness, presence of meaning, search for meaning, mindfulness, or smartphone addiction.

Years listening to podcasts was negatively associated with smartphone addiction. Podcasts per month was positively associated with presence of meaning. Lastly, parasocial relationships were positively associated with relatedness, and social engagement was positively associated with presence of meaning.

## Discussion

The aim of this study was to identify dispositional predictors of podcast listening and examine the associations between aspects of podcast listening, dispositional predictors, and psychological outcomes. We found that several individual difference variables predicted podcast listening. As predicted, we found that people who were higher in openness to experience, interest type epistemic curiosity, and need for cognition were more likely to have listened to a podcast. This indicates that those who listen to podcasts have stronger informational needs. These findings are consistent with past research which found that openness, curiosity, or need for cognition was associated with use of other new technologies [20,25] and using online platforms for informational purposes [14,27,28]. Contrary to predictions, participants who were higher in need to belong were less likely to have listened to a podcast. Furthermore, when we examined other Big Five factors, we found that participants who were higher in neuroticism were less likely to have listened to a podcast. These two findings help to differentiate podcast listening from social media use, which is positively associated with the need to belong [32] and neuroticism [21]. Lastly, an examination of demographic predictors revealed that men were more likely to have listened to a podcast than women. This is consistent with other studies that have found that similar gender differences in podcast listening [11,34,66]. It could be due in part to broader gender differences in technology use whereby women place greater value on connectivity and men place greater value on information [67]. Together, these findings suggest that informational needs rather than social or emotional needs may be more relevant motivations for podcast listening. This is consistent with past findings that information was a more strongly endorsed motive for podcast listening than other more social motives among regular podcast listeners [11].

Additional analyses revealed that social aspects of podcast listening were related to personality and other aspects of listening. We found positive associations between extraversion and social engagement with podcasts, and between agreeableness and parasocial relationships with one’s favorite podcast host. These findings are consistent with our understanding of extraversion and agreeableness as involving sociability and a communal orientation, respectively [18]. Social aspects of listening were also related to exposure to podcasts and hosts. Both social engagement with podcasts and parasocial relationships with one’s favorite podcast host were stronger among those who spent more time listening to podcasts. Additionally, social engagement was associated with listening to more podcasts per month and parasocial relationships were associated with listening to more podcasts with hosts they knew from somewhere else. These findings are consistent with past research which found positive associations between media consumption and both social engagement [8] and parasocial relationships [36,40].

We found no support for the prediction that greater time spent listening to podcasts would predict greater autonomy, competence, relatedness, and meaning, lower mindfulness, and greater smartphone addiction. However, we did find that presence of meaning was higher among those who listened to more podcasts per month and were more socially engaged with podcasts. Furthermore, parasocial relationships were associated with a greater sense of relatedness. These associations extend prior work on the potential gratifications of podcast listening [8–11]. The relatedness finding is consistent with past research which has found that entertainment media can satisfy basic psychological needs [41] and that parasocial relationships can meet belonging needs [37]. The meaning findings could stem from several factors, including the satisfaction of basic psychological needs, positive affect, curiosity, coherence, and narrative elements [47–50]. Future research should further examine the processes through which socially engaging with podcasts and listening to more podcasts could enhance meaning.

We also found that smartphone addiction was lower among those who had been listening to podcasts for more years. More seasoned listeners might have begun listening to podcasts on other devices such as an iPod [4], and thus, might be less dependent on their smartphone for podcast listening. We also found that age was positively associated with years spent listening to podcasts. Thus, unlike past studies which found that younger people listened more intently [11], we found that that older people had a longer listening history, suggesting different demographic profiles of new and seasoned listeners.

### Limitations

A limitation of this study is that the cross-sectional nature of our design does not allow us to draw causal conclusions. Further limitations are associated with our measures. Participants might have been inaccurate when reporting their podcast use and the individual difference measures might not have adequately assessed the constructs of interest. Future research could use a daily diary methodology to track podcast use over shorter timeframes to improve the accuracy of reporting and use alternate measures of predictors and outcomes to further expand our understanding of the psychology of podcast listening.

### Conclusions

Our findings extend our knowledge of podcast listening by demonstrating that across a broad global sample, people who have listened to a podcast have higher informational needs, lower belonging needs, and lower neuroticism than those who have not. This helps to contextualize motives among regular podcast listeners and demonstrates novel behavioral outcomes associated with commonly assessed individual differences. Among our podcast listeners, we found that socially relevant personality characteristics were associated with social engagement with podcasts and hosts. Furthermore, we provide some initial evidence that listening to more podcasts and socially engaging with podcasts are associated with greater presence of meaning and forming parasocial relationships with hosts is associated with a greater sense of relatedness.

Our findings support the idea that podcasts can provide informational and social gratifications to listeners [9–11]. Through our inclusion of participants who had not listened to podcasts, we were able to gain greater insight into dispositions that motivate podcast use separate from our examination of aspects of podcast listening and obtained gratifications. We conclude that informational needs likely motivate podcast listening and that certain types of listening can provide social gratifications.
